# Incidence of vertebral artery injury in patients undergoing cervical spine trauma surgery in correlation with surgical approach: A review

**DOI:** 10.1097/MD.0000000000034653

**Published:** 2023-09-15

**Authors:** Evangelos Sakellariou, Ioannis S. Benetos, Dimitrios-Stergios Evangelopoulos, Athanasios Galanis, Fani Alevrogianni, Michail Vavourakis, Vasilios Marougklianis, Georgios Tsalimas, Spiros Pneumaticos

**Affiliations:** a 3rd Department of Orthopaedic Surgery, National & Kapodistrian University of Athens, KAT General Hospital, Athens, Greece; b Department of Anaesthisiology, KAT General Hospital, Athens, Greece.

**Keywords:** cervical spine, cervical spine fusion, spinal cord injuries, vascular injury, vertebral artery injury

## Abstract

Spinal cord injuries at the cervical spine level represent the most consequential of the related injuries at all levels of the spine. They can trigger permanent unilateral or bilateral damage with conspicuous disability. Regarding unstable injuries, the gold standard approach is open reduction and osteosynthesis, which can select between anterior and posterior surgical access. Each of the aforementioned approaches demonstrates both advantages and disadvantages; thus, it is up to the surgeon to determine the optimal option concerning the patient’s safety. Diligent intraoperative control of anatomical reduction is pivotal to obtaining the best feasible postoperative outcomes. Literature data delineate copious complications following surgical intervention in the cervical spine. Indubitably, the most crucial intraoperative complication accounts for vascular injuries, with the most preponderant being the corrosion of the vertebral artery, as it is potentially life-threatening. This paper aims to provide a succinct and compendious review of the existing literature regarding cervical spinal cord injuries and to deduce many inferences concerning the incidence of iatrogenic vertebral artery injuries in relation to the surgical approach for fracture reduction.

## 1. Introduction

Depending on the approach, cervical spine (CS) surgery is associated with multiple intraoperative complications. The anterior approach is correlated with dysphagia, esophageal injury, paresis of laryngeal nerves, and Horner syndrome. Meningeal erosion, epidural hematoma, nerve root injury, and spinal cord infarction are chiefly prompted by posterior cervical spine approaches. Additionally, intraoperative vascular injuries have been reported in both surgical approaches, and whilst infrequent, they feature significant mortality (7.22%). This figure is augmented steeply if other nonvascular complications are also present. Blood vessels exposed during an operation are the vertebral artery, carotid artery and its branches, thyroid arteries, and internal jugular vein.^[1]^

Intraoperative vertebral artery injury is the most recurrent vascular injury in cervical spine surgery, accountable for potentially devastating complications, exhibiting rates as high as 86.6% compared to other vascular injuries.^[1]^ The overall incidence of iatrogenic vertebral artery injury is mercifully rare (0.8–1.4%), with percentages that vermiculate according to the surgical approach employed. More specifically, posterior access at the C1–C2 level leads to vertebral artery injury in 4.1% to 8.2%, while the anterior approach accounts for 0.3% to 0.5% of iatrogenic vertebral artery injuries.^[1]^ Intraoperatively, the highest rates of iatrogenic vertebral artery injuries occur during vertebral drilling (20.6–61%) or placement of surgical instruments (16–31.44%). Less commonly, it can be provoked during soft tissue manipulation, detachment of the ossified posterior longitudinal ligament or by using diathermy and manipulations nearby the vertebral artery.^[1]^ The sorts of spinal column vascular damage are classified as arterial rupture, which is the most frequent form (41.24%), dissection (5.67%), pseudoaneurysm formation (16.49%) or arteriovenous fistula (2.58%), thrombosis, embolism or even complete blockage (4.64%).^[2]^

The clinical presentation of patients sustaining vertebral artery injury varies from being utterly asymptomatic to hypovolemic shock, depending on the graveness of the injury, irrespectively of neurological complications.^[2]^ Principal clinical findings of iatrogenic vertebral artery injury include intraoperative bleeding accompanied by hypotension and tachycardia, posterior circulation infarction, neck edema, dyspnea, impaired level of consciousness, or in more acute cases, hemorrhagic shock and even death. Most symptoms linked to vertebral artery injury are precipitate, although late-onset symptoms may appear due to delayed bleeding complications, ruptured pseudoaneurysm or arteriovenous communication.^[1]^

Posterior circulation infarcts are characterized by atypical symptoms, such as unsteadiness, vertigo, diplopia, cortical blindness, alternating paresthesia, tinnitus, dysphasia, dysarthria, tetraplegia or ataxia. In addition, late-onset ischemic complications may be related to embolism due to partial occlusion or destruction of the vertebral artery. Predominately, the left vertebral artery is dominant in diameter, and although intraoperative identification is more straightforward, enhanced attentiveness is required during manipulations. Notably, the posterior inferior cerebellar artery is a branch of the vertebral artery, and vertebral artery injury at this level triggers a lateral cord infarction.^[1]^

Restorative surgery to the cervical spine comprises 2 main approaches, anterior and posterior, with discrete techniques, the indications of which are perused below. The anterior approach is the most broadly used and exposes the vertebral bodies from C2 up to T1. The main indications involve patients suffering from pathological conditions that lead to cervical myelopathy. For the surgical treatment of burst fractures of the lower CS (C3–C7) with spinal cord compression, anterior access and spinal fusion are regarded as the gold standard method. On the other hand, the only case in which this particular approach would be preferred to treat fractures of the upper part of the CS is for type II fractures of the odontoid process of the axis (C2) because it offers innocuous reduction and stabilization.^[3]^ Selection between left and right anterior cervical access relies on the training and preference of the surgeon. Nonetheless, literature evidence indicates that the right anterior approach is superior to the left as it exposes the recurrent laryngeal nerve directly in the surgical field, diminishing the risk for injury. For single-level restoration, the incisions are transverse, whereas, in multi-level surgeries, longitudinal incisions are favored.^[3]^ An incision is made in the skin and subcutaneous tissue, and entry is made through the muscularis platysma. Deep cervical fascia is identified and traversed along the anteromedial border of the sternocleidomastoid muscle. After palpating the carotid pulse, the incision is deepened on the inside of the common carotid. At the same time, the trachea and esophagus are pushed towards the midline, in the direction of the prevertebral fascia, which is cut longitudinally, providing instant access to the vertebrae.^[3]^

The posterior approach should be employed in CS injuries with dislocations of the apophyseal joints or in injuries of the posterior ligamentous system, as the posterior longitudinal ligament is the important stabilizing mechanism impeding the overflexion of the CS. Posterior approaches are utilized for laminectomy and laminoplasty and are indicated for posterior spinal canal decompression in the absence of anterior compression. Traumatic injuries of the upper part of the CS, such as type II/ III fractures of the odontoid process of the axis, atlantoaxial instability with or without rotational displacement, and unstable Jefferson fractures, are approached posteriorly. With regards to the lower part of the CS (C3–C7), the posterior approach is preferred when a significant degree of damage to the posterior elements is evident, when reduction cannot be accomplished either by a closed method or by anterior approach, and finally in cases where anterior pressure of the spinal cord or herniated intervertebral disc are absent.^[4]^ The patient is placed in a prone position, with the head immobilized, and the cervical part is exposed with slight flexion and head extension. The skin incision is carried out on the mid-posterior surface of the neck, the length of which depends on the number of levels of surgical interest. Identification of the medial posterior surface is done under fluoroscopic control. The deep course to the spine follows the mid-cervical line, as it is avascular, to the spinous processes of the cervical vertebrae. This is followed by the subperiosteal detachment of the cervical muscles in a cephalocaudal direction, with complete detachment of the spinous processes, the vertebral arches and part of the articular processes from C3 to C6. Punctilious hemostasis is required in the interosseous spaces, protecting the interosseous fat and avoiding damage to the facet joints. A special expander is placed without much tension on the muscles, and the vertebral arches and spinous processes are fully exposed.^[4]^ The conventional surgical decompressive approach to CS is the cervical laminectomy. Technological advancements, with the introduction of the surgical microscope and new surgical tools, have pronouncedly decreased the postoperative complications of laminectomy and ameliorated its long-term results. Cervical laminoloplasty is a modified posterior laminectomy.^[4]^

Contemporary literature findings concerning CS surgery do not provide explicit evidence regarding the type of surgical approach and intraoperative damage to the vertebral artery. Preoperative preparation and visualization of the vascular anatomy of the area are of cardinal significance. Surgeons tend to choose the approach with which they are most familiarized and more appropriately trained. However, in case of complications, expeditious diagnosis and treatment are imperative concerning major vascular injuries with pernicious consequences for the patient. The purpose of the present study was to investigate whether there is a substantial difference regarding the incidence of vertebral artery injury in cervical spine operations depending on the type of surgical approach, posterior or anterior.

## 2. Materials and methods

A systematic literature review was executed, the online databases scrutinized to identify scientific articles on the subject were: Pubmed – NCBI, Web of Science, Cochrane Library, Scopus and Embase. In addition, a separate search was conducted in the archives of the following medical journals: Spine, European Spine Journal, and Journal of Bone and Joint Surgery. Clinical information searched involved studies with anterior or posterior access in patients undergoing cervical spine surgery, focusing on the complication of intraoperative vertebral artery injury. Data included operative time, blood loss during surgery, the height of the vertebral column of the CS, intraoperative techniques, the diagnostic algorithm for suspected vascular injury and its management, as well as the overall risk of each treatment technique. No restrictions were implemented regarding the scientific articles’ language and date of publication. Articles available in full text were studied to retrieve additional studies relevant to our topic.

Keywords (mesh terms) employed during the search were: cervical spine injuries, spinal cord injuries, rehabilitation, vertebral artery atrophy, vascular injury, vertebral artery injury, anterior cervical spinal fusion, posterior cervical spinal fusion. All studies analyzed the type of approach (anterior, posterior, or a combination of the 2) chosen in relation to the type of spinal injury. The type of osteosynthesis utilized, total time of surgery, and the corresponding incidence of vertebral artery injury was perused. In all studies, when a vascular injury was suspected, patients underwent imaging and were treated surgically, either by open access, local hemostasis, or an endovascular technique. From the rigorous literature review, 35 articles were included that met the above criteria.

**Figure 1.**
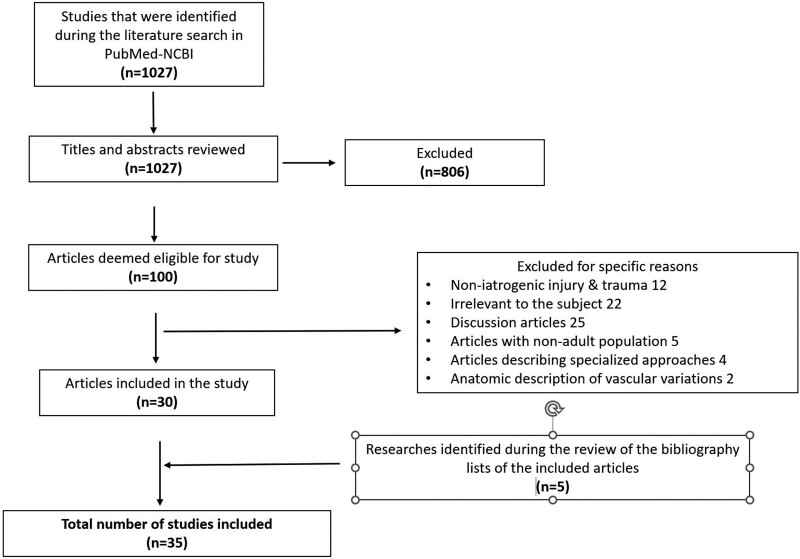
Study’s flowchart according to the PRISMA guidelines.

Inclusion criteria involved all published studies where the patients: 1: did not suffer from other serious underlying diseases. 2: Had damage at the C1–C7 level and underwent surgery. 3: Had suffered an injury to the CS with neurological compression. 4: Surgical technique was performed by an anterior or posterior approach, or a combination of the 2, and 5: mean age was between 18 and 65 years. We excluded all published studies, which: 1: involved children. 2: Included animals. 3: Were discussion articles or clinical case reports. 4: Involved conservative treatment. 5: Thoracic spine injuries also coexisted, 6: spinal inflammation or tumor coexisted, and 7: patients with coexisting vascular injury preoperatively. From the rigorous literature review, 35 articles were included that met the above criteria (Fig. 1).

## 3. Results

### 3.1. Anterior approach

#### 3.1.1. Retrospective studies.

In 2017, in a Greek retrospective study^[5]^ analyzing complications associated with the anterior approach in cervical spine surgery in 114 patients, no incident of iatrogenic vertebral artery injury was reported, promoting the abovementioned as a safe method.

#### 3.1.2. Prospective studies.

Neo M et al^[6]^ in 2008 carried out an observational study of 5600 procedures involving anterior cervical decompression and posterior placement of transarticular spinal fusion screws, investigating the incidence of iatrogenic vertebral artery injury. The rate of vascular damage was 0.14% (8 out of 5641) for the anterior and 1.3% for the posterior approach, respectively. For cases of vascular injury, bleeding was controlled by local packing. No deaths or neurological complications were recorded. They concluded that surgeon experience was a salient factor, although careful preoperative assessment confines complication rates.

A systematic literature review in 2005 by Inamasu J et al^[7]^ accounted the incidence of anterior approach vertebral artery injury about 0.3% to 0.5%. Promptitude to prevent intraoperative bleeding and preoperative vascular anatomical knowledge was promoted as the main protective factors. They accentuated, however, that vertebral artery injury can escape from attention, and in high suspicion, early diagnosis is vital due to unpredictable complications in asymptomatic patients. Yet, in cases of non-compensating collateral circulation, patients can suffer vertebral-basal ischemia that can be fatal. Peng CW and colleagues 2009^[8]^ performed a systematic review of vertebral artery injury in cervical spine surgeries and analyzed anatomic theories, alternative management methods, and measures that protect from iatrogenic vascular injury. Anterior spinal fusion exhibited a lower risk, while the presence of an anatomic abnormality elevated the risk of iatrogenic vascular injury. Labelling and intraoperative guidance based on guiding points were featured to protect against vascular erosion. Finally, preoperative computed tomography imaging was deemed indispensable, and in case of vascular damage, endovascular repair was indicated as the preferred method.

In 2017, Guan Q et al,^[9]^ reviewing 25 articles with a total of 54 patients sustaining iatrogenic vertebral artery injury in anterior access operations, inferred that intraoperative aggravating factors are extensive lateral decompression, loss of landmarks, anatomical variations or vertebral artery pathology. Concerning the surgical steps, drilling in 61%, material placement (16%) and soft tissue removal (8%) were responsible for vascular injury.

In the same year, Lo WB and colleagues,^[10]^ in their literature review, examined the cases of late bleeding after iatrogenic vertebral artery injury in anterior cervical discectomy. They primarily reported that iatrogenic injury is infrequent but potentially life-threatening. Additionally, they pointed out that despite occlusion of bleeding and an initial normal angiography, late aneurysm and sectioning of the artery may reoccur. Hence, emergency angiography should be re-executed even if the first was normal. Moreover, vascular reconstruction should be performed instantaneously. Assuming, therefore, that the contralateral vertebral perfusion is intact, intravascular embolization provides a safe and fruitful solution to the immediate restoration of the vascular patency. They emphasized that bleeding after cervical discectomy and fusion (technique) is customarily of venous origin, and surgical drainage of the hematoma is highly recommended. However, surgical decompression carries a high risk of exsanguination in the adverse event of postoperative bleeding in a patient with vertebral artery injury. Therefore, the patient must undergo emergency intubation, followed by definitive treatment. Grounded on the above, they propounded that definitive treatment of vertebral artery injury should be determined on a case-by-case basis due to the rarity and intricacy of the vessel and anatomical variations.

In 2018, Daniels AH and colleagues^[11]^ evaluated the unpropitious events associated with an anterior approach in cervical spine surgeries and concluded that procedures such as total disc replacement, and not using a morphogenic protein of bone as an adjunct to fusion do not raise significant concerns. Thus, apposite strategies must be employed to avoid adverse events, and the treating physician must recognize, detect and manage such events when they transpire.

Two years later, Yee TJ and colleagues^[2]^ analyzed the complications of the anterior surgical approach of the cervical spine in a systematic literature review of 240 studies from the last 30 years. The abovementioned approach was associated with iatrogenic injury of the vertebral artery at a rate of 0.4% (0.2–2.2%), giving prominence to its lower morbidity. In terms of management, they described that both endovascular repair and local surgical hemostasis are efficacious methods and tantamount concerning postoperative neurological deficit. They studied the surgical stage with an increased risk of iatrogenic vascular damage and pointed out extensive decompression with drills. Finally, like previous authors, they underlined the momentousness of preoperative vascular imaging as a protective measure.

### 3.2. Posterior approach

#### 3.2.1. Retrospective studies.

As early as 1995, in a retrospective study of 78 patients undergoing posterior approach cervical spine surgery, Heller JG et al^[12]^ recorded complications from the insertion of 654 screws, 8.4 per patient. They reported no vascular complication rates corresponding to the number of screws inserted. In fact, they recalled that a cadaver specimen study had predicted certain rates of anatomic complications associated with lateral screw insertion. However, this particular study observed that the risk of lateral screw insertion is glaringly lower than predicted in vitro.

In 2011, the surgical group of Dorward IG^[5]^ retrospectively reported their clinical experience through 103 patients in whom a C2 transplate screw was placed posteriorly and reviewed the existing literature. They pointed out that their outcomes were relevant to existing data, as 95% of the patients did not experience vascular complications, and the employment of a 3.5 mm diameter screw was safe.

#### 3.2.2. Prospective studies.

In 2008, Menezes AH^[13]^ performed a prospective study of 25 patients regarding postoperative care and complications after a posterolateral-longitudinal transcondylar approach to the ventral foramen and superior cervical spinal canal. He accented that the technique mentioned above is versatile. Exposure of the vertebral artery is sufficient, with little or no retraction of crucial neurovascular structures in the area, making it a safe technique.

#### 3.2.3. Meta-analyses.

In 2019, Lvov et al^[14]^ executed a meta-analysis on the risk factors of structural screw placement at the C1–C2 level with posterior access. They reported that concerning the period 1989 to 2018, the risk of iatrogenic vertebral artery injury was 1.7–1.8% per screw placement, whilst regarding the period 2011 to 2018, the risk was remarkably minimized (0.3%). Patients younger than 55 years of age, with degenerative spinal lesions or anatomical variations of the vertebral artery, in whom skeletal traction was placed for reduction, or where C1–C2 reduction was incomplete, demonstrated increased incidence of symptomatic vascular damage. Other surgical characteristics of enhanced risk were the large incision size, emergency surgery, extensive drillings and placement of bone grafts. Placing a patient in a HALO or Mayfiled device revealed no difference in terms of vascular complications, while the use of lateral fluoroscopy was associated with an increased risk of vertebral artery injury.

In 2021, Gaith AK and his colleagues^[15]^ in a meta-analysis of 773 patients who underwent cervical spine surgery with posterior approach, concluded that iatrogenic vertebral artery injury is a rare complication. Specifically, they reported that in posterior spinal fusion at the C1–C2 level, the rate of vertebral artery injury is 2% and it increases by 1% for each additional screw placed. According to their review, they also observed that anatomical vertebral artery abnormalities is the most prevalent predisposing factor for iatrogenic injury.

#### 3.2.4. Systematic literature reviews.

In 2005, Inamasu J et al^[16]^ reviewed the literature for the most pertinent screw-repair method of craniocervical instability, employing a posterior surgical approach. Transarticular screws at the C1–C2 level, regardless of the underlying cause of instability (trauma, congenital lesion, tumor, degeneration), were regarded as the gold standard method. Atlantoaxial structural fixation with screws, bone grafts, and wires featured a high rate (4.1%) of vascular injury during transosseous screw entry between the transverse processes. Therefore, preoperative computed tomography imaging to avoid misplacement of screws was reckoned as requisite. According to findings, C1 lateral mass screw placement had a lower incidence of iatrogenic vertebral artery injury. Contrariwise, atlantoaxial or structural screws could not reach a firm conclusion due to the lack of cases and the reduced stability of the method. Nonetheless, they argued for their limited vascular injury risk. Finally, in terms of occipital stabilization, mechanical axis correction should be avoided due to reduced stability.

### 3.3. Anterior and posterior approach

#### 3.3.1. Retrospective studies.

In 2012, a review of 39 cases of vertebral artery injury^[17]^ illustrated the relationship between vertebral artery injuries and post-injury management for avoiding neurological complications. Initially, the incidence of iatrogenic vascular injury was reported to be 0.3% to 0.5%, and it was emphasized that the preferred method of dealing with bleeding was local packing with hemostatic gauze or immediate anastomosis and endovascular repair to prevent late complications. In fact, in 76.4% of these patients, no neurological deficit was observed, and only 17.9% of the cases presented postoperative neurological complications. Also, there was a 2.6% mortality reported due to uncontrolled bleeding. However, swift repair did not seem to hinder neurologic damage, while on the contrary, hemostatic agent packing featured an elevated risk for postoperative bleeding, pseudoaneurysm and arteriovenous communication. Embolization or ligation deteriorated the risk of postoperative neurological complications. Thus, the authors deduced that vessel ligation should be a primary treatment method. Notwithstanding, they accentuated that in case of vertebral artery injury during drilling, bleeding should be controlled by local packing with a hemostatic agent, suturing and reconstruction of the vessel, and then endovascular repair. In case of failure to restrain bleeding, intraoperative endovascular embolization and ligation should be pondered, but with the peril of neurologic complications.

In 2013, Steltzlen C et al,^[18]^ in a retrospective study of 22 cases with unstable odontoid process fractures following surgical repair, presented the risk of iatrogenic vertebral artery injury at a rate of 1.3% to 5.8%. They also underlined the importance of preoperative planning regarding C1–C2 arthrodesis in conjunction with sedulous cognizance of vertebral artery anatomy. In a retrospective study, Wewel JT and colleagues in 2019^[19]^ described perioperative complications in anteroposterior cervical spine approach for multilevel repair, and vertebral artery injury was observed in 1.4% of the cases.

#### 3.3.2. Meta-analyses.

In a 2015 meta-analysis, Elliot RE et al^[20]^ included 1471 patients with C1 massa lateralis screw placement and 2905 with C1 posterior arch screw and concluded that although they did not differ strikingly in terms of neurological effects, due to an increased risk of vascular erosion in erroneous screw placement, sacrificing the C1 root is more expedient.

Five years later, Klepikowski T and colleagues^[21]^ scrutinized the incidence of descending vertebral artery in terms of its anatomic variations and contrasted the type of surgical access with the patient’s outcome. Type of access, preoperative assessment and vertebral artery course, preponderantly at the level of C2, determine the choice of insertion site for transverse screws. Anatomical variations, with the highest incidence of descending vertebral artery, are of uppermost outcome consequence. In addition, they denoted both anterior and posterior approaches as similar techniques in the absence of anatomical variations.

In 2022, Soliman MAR et al^[22]^ carried out a meta-analysis including 1768 patients and 8636 spinal fusion screws and investigated the complications correlated with subaxial placement of translaminar versus lateral screws in the cervical spine, showing no differences between them. In the same year, Chang MC group^[23]^ performed a meta-analysis of 5101 publications comparing transarticular screw fixation versus segmental screw-rod fixation at the C1–C2 level, leading to segmental spinal fusion exhibiting a lower risk of vascular complications.

#### 3.3.3. Systematic literature reviews.

In 1998, Wright NM, as a member of the American Society of Neurosurgeons,^[24]^ examined the risk of vertebral artery injury during C1–C2 structural screw placement, which translated into a figure of 4.1% for each patient and 2.2% for each screw placement. In 2007, Bruneau M and colleagues^[25]^ delineated surgical indications and techniques for multilevel oblique somatectomies. They relied on a much older study from 1992, which emphasized that the above technique is quite exacting and demands preoperative control of the vertebral artery. Consequently, they presented the anterior approach as an alternative option, as it demonstrates fewer osteotomies and provides wide anterior decompression and somatectomy. In 2010, Lall et al^[26]^ conducted a literature review regarding the complications of spinal fusion of the craniocervical joint. The results displayed a range of 1.3% to 4.1% damage to the vertebral artery during the placement of C1–C2 transarticular screws, with an increased risk in the case of descending vertebral artery. They inferred that painstaking preoperative knowledge of anatomical variations is the most essential factor in preventing iatrogenic vascular damage. In 2013, Coe JD and colleagues^[27]^ performed a systematic literature review concerning cervical spine lateral screw fixation, reporting no vertebral artery injury linked with the above technique for the subaxial spine, contrasted to the upper cervical spine.

In the same year, Yoshihara H et al^[28]^ conducted a systematic literature review of the complications of screw placement in the subaxial spine using lateral versus translaminar screws at the C3–C7 level. Translaminar screws reported a high rate of vertebral artery injury compared to lateral ones, which, however, in terms of stabilization and complications, appeared to be commensurate with vascular injuries. Like previous authors, they inferred that in-depth anatomy knowledge requires a personalized preoperative plan and hampers iatrogenic injury of the vertebral artery. In 2016, Cheung JP et al^[29]^ inspected the complications of anterior and posterior approaches in cervical spine surgeries. Regarding anterior surgical access, iatrogenic vertebral artery injury was observed in 0.3% to 0.5% of the cases when decompressing the vertebrae more than 18–20 mm or when the decompression included the C3–C4 level or when C7 presented pathology. Concerning the posterior approach, the risk was palpably enhanced (1.3–4%), specifically during the placement of C1–C2 transarticular screws, lateral decompression or penetration into the foramen. In 2019, following a literature review, Wangqin R and colleagues^[30]^ proposed the utilization of a covered stent to restore iatrogenic vertebral artery injury in unconstrained bleeding. They suggested that the covered stent for intraoperative vessel repair, because of being wrapped by the membrane, can unreservedly seal the tear and impede bleeding. Although vertebral artery injury is an unusual but critical complication, intraoperative endovascular repair with portable fluoroscopy is justified and attainable, allowing completion of the procedure.

In 2021, Turgut M et al,^[31]^ in a review of 72 articles with 194 cases (1962–2021) of vascular injury deriving from iatrogenic injury in both anterior and posterior approaches, demonstrated that anterior screws for odontoid process fractures, 4-level cervical discectomy and fusion, and extensive drilling were connected with a soared risk of intraoperative vascular injury. They reported that surgeon disorientation and loss of landmarks, combined with anatomical abnormalities, augment the risk of injury, resulting in massive bleeding, which is exceedingly arduous to control. Preoperatively, they pinpointed the contribution of computed tomography, magnetic resonance imaging, magnetic resonance angiography, or conventional angiography. Endovascular repair should be the proper treatment method. Regarding posterior access, atlantoaxial structural screw fixation was ranked as the most hazardous technique, with a vascular injury rate of 8%, and was therefore abandoned. Alternatively, C1 lateral mass screw placement and C2 transpendicular screw placement were employed. In subaxial lateral mass screw placement, 0.6% of vertebral artery injury was observed, with an even lower risk for cervical foraminectomy, petalectomy, or petaloplasty. Anatomically, the level at which iatrogenic vertebral artery injury was predominantly observed were C1–C2 and C7. An additional risk was added with the existence of vascular variations, chiefly in the presence of bifurcation or angulation. They concluded, nonetheless, that early diagnosis and intervention are consequential regardless of the approach.

In 2021, An TY et al^[32]^ reviewed the literature on iatrogenic vertebral artery injury and observed increased intraoperative risk during vertebral exposure, decompression, or instrument insertion. Yongjun T and colleagues, in 2022^[33]^ reviewing literature regarding late bleeding owing to vertebral artery injury in ACAF technique, underlined that intraoperative local packing with bone graft for bleeding control is the optimum method. They also advocated performing preoperative imaging to discern possible vascular variations and calculate the distance from the transverse foramen and its relationship with the posterior longitudinal ligament. In 2022, Yi HJ^[1]^ performed a systematic literature review with critical parameters for the epidemiology and management of iatrogenic vertebral artery injury in cervical spine surgery. Corrosion of the vertebral artery exhibited the highest percentages (41%) as a type of vascular injury, followed by pseudoaneurysm formation at 16.4%, dissection at 5.67%, embolism at 4.64% and, more scarcely, arteriovenous communication (2.58%). Anterior surgical access presented low rates of iatrogenic vascular injury (0.3–0.5%), with expandable drilling during discectomy, osteosynthesis plate placement with off-midline screws, tumors and infections being aggravating factors. On the contrary, in terms of posterior access to the C1–C2 level, intraoperative vertebral artery injury was recorded at a rate of 4.1% to 8.2%. It was also pointed out that there is dichotomy regarding the most appropriate method of treating intraoperative iatrogenic damage to the vessel. As alternative options, bleeding control with the primary goal of avoiding vertebral-basal ischemia and preclusion of its complications, immediate packing with hemostatic gauzes, microvascular repair or ligation of the vessel, anastomosis, and endovascular repair was described. A dominant finding was that preoperative anatomical knowledge of vertebral artery’s origin, course, and variations diminish rates sharply, irrespective of access.

## 4. Discussion

Cervical spine surgery is irrefutably one of the most onerous surgeries in Orthopedics, providing quality of life to the patient, preventing permanent paralysis and relieving him/her of pain. However, access to the cervical vertebrae requires prowess, as it carries potentially fatal complications due to its proximity to major neurovascular structures. The most life-threatening complication is the iatrogenic injury of the vertebral artery, which is the subject of the current review. Regarding types of damage, the most common is intraoperative erosion, in a range that reaches 41%, which can be detrimental. However, being usually innocent as more than 75% of patients do not present neurological symptoms postoperatively.^[34]^ The second most frequent form of damage is pseudoaneurysm formation (16.49%), while cases of dissection (5.67%), vascular embolism (4.64%), and formation of arteriovenous fistula (2.58%) have been documented.^[1]^ The vertebral artery is renowned for being subject to a profusion of anatomical variations, and therefore preoperative knowledge of its course is vital for safe access to the cervical vertebrae, avoiding vascular injury.^[35]^ Nevertheless, the presence of anatomical variations is correlated with an augmented risk of iatrogenic damage.^[8]^ According to literature data, the variant with the utmost risk of iatrogenic injury is detected near the point of its emergence if the vessel demonstrates increased angulation or bifurcation^[31]^ or involves the equine vertebral artery.^[26]^ As claimed by Yongjun T et al, who studied the incidence of iatrogenic injury in spinal fusion with the ACAF technique, besides perceiving the anatomical variation of the vessel, the calculation of the distance of the vertebral artery from the transverse foramen and its relationship with the posterior longitudinal ligament is of momentous gravity.^[33]^ Although literature accentuates the essential requirement for preoperative knowledge of the anatomy and course of the vertebral artery, the imaging technique of choice remains contentious. It is even worth mentioning that according to Tan La et al, preoperative ignorance of vertebral artery anatomy is associated with an upraised incidence of injury at a rate of 35%.^[36]^ In most studies, computed tomography with intravenous contrast is adequate, although there have been reports that consider digital angiography more reliable.^[31]^ In accordance with the most present-day literature data by Yi HJ,^[1]^ who examined the epidemiology of iatrogenic vertebral artery injury in cervical spine surgeries depending on surgical approach, the posterior approach is regarded more perilous for vascular injury, at a rate of 4.1% to 8.2%. Turgut M et al deduced that during the posterior approach, repair at the level anteriorly of C7, laterally of C3–C7, and posteriorly of C1–C2 is characterized by an increased risk of vascular injury.^[31]^ Anterior access is associated with a much smaller percentage of iatrogenic damage to the vessel (0.3–0.5%), although technically more grueling.^[1]^ In a systematic literature review by Yee TJ et al, anterior access, apart from a lower risk of iatrogenic injury, is also linked to lower patient morbidity rates.^[2]^ It is widely established that the incidence of injury depends on the number of osteosynthesis screws, regardless of the access.^[12]^ Moreover, the type of screw is also related to the incidence rate, although it is not evident whether any type is superior in terms of stabilization of the subaxial spine. In fact, transverse versus lateral screws are more feared for iatrogenic vertebral artery injury.^[28]^ The screw diameter also plays a crucial role, according to a Greek review of the literature, with a diameter >3.5 mm increasing the risk of vascular damage.^[37]^ Among the anterior approach surgical techniques, anterior cervical discectomy and fusion are literature-wise considered to be the safest technique in terms of vascular complications.^[16]^ Yee TJ et al investigated the complications of anterior access concerning the incidence of vertebral artery injury, emphasizing that multilevel (mainly 4) spinal fusion repair is associated with an increased risk. Analyzing surgical steps, the stage in which decompression drills are performed is blamed for vascular injury.^[2]^ In contrast, despite the restoration of multiple levels of spinal fusion when oblique somatectomies are executed, the risk of iatrogenic injury is decreased due to wide exposure of the vertebral artery in the surgical field.^[25]^ A recent meta-analysis concerning posterior access spinal fusion at the C1–C2 level inferred that the risk of vascular injury overall was 2% and even increased by 1% for each additional screw placed.^[15]^ This rate was markedly lower than a 1998 prospective study, in which the risk of vascular injury was 4.1% and increased by 2.2% for each additional screw placement.^[14,24]^ The abovementioned discrepancy can be presumably attributed to the improvement of surgical techniques and the advances in intraoperative imaging methods, whilst the overall incidence of vertebral artery injury over time accounts for 1.7% per screw.^[14]^ Lall R et al reviewed literature data on craniocervical fusion surgery and found the risk of iatrogenic vertebral artery injury to be 1.3–4.1%.^[26]^ Contrarily, the risk of vascular damage is reduced when the access is converted to posterolateral craniocervical.^[13]^ In a systematic literature review conducted by Park HK et al, the management methods of iatrogenic vertebral artery injury in anterior access consisted of simple local management of bleeding with hemostatic gauzes, acute vascular repair by suturing the vessel and restoring its patency, or immediate endovascular repair postoperatively.^[8]^ No technique supremacy was discovered, while the common goal was preventing late complications and neurological damage. It is imperative to accentuate that only roughly ¼ of the patients who experience iatrogenic vascular damage and are treated effectively will suffer a neurological deficit postoperatively.^[8]^

Even with normal postoperative angiography, local patching for bleeding control did not expunge the possibility of late bleeding recurrence. In fact, in cases where exigent surgical decompression of the hematoma was necessary, surgical risk increased dramatically as it was associated with extensive blood loss. In conclusion, intravascular embolization was preferred as the safest option compared to other methods, provided that the contralateral vertebral artery is patent, avoiding vertebral-basal ischemia.^[9]^ Even more recent literature data corroborated that the employment of endovascular-covered stents is capable of sealing vascular erosion and restoring the patency of the vertebral artery, enabling the prosecution of spinal fusion surgery.^[7]^

## 5. Conclusion

Iatrogenic vertebral artery injury during cervical spine surgery is an infrequent but potentially pernicious complication that can be fatal. Meticulously reviewing the literature, comparing anterior and posterior surgical access promotes the anterior as safer regarding the incidence of vascular injuries. Nonetheless, selecting the approach where the attending surgeon is best trained is considered more expedient. Apposite preoperative preparation with imaging anatomical variations of the vertebral artery is of paramount significance to diminish the rates of intraoperative vascular complications.

## Author contributions

**Conceptualization:** Evangelos Sakellariou, Ioannis S. Benetos.

**Data curation:** Vasilios Marougklianis, Georgios Tsalimas.

**Investigation:** Fani Alevrogianni, Michail Vavourakis.

**Methodology:** Dimitrios-Stergios Evangelopoulos.

**Supervision:** Spiros Pneumaticos.

**Visualization:** Athanasios Galanis.

**Writing – original draft:** Evangelos Sakellariou.

**Writing – review & editing:** Ioannis S. Benetos.
